# Human *Plasmodium knowlesi *infections in young children in central Vietnam

**DOI:** 10.1186/1475-2875-8-249

**Published:** 2009-10-30

**Authors:** Peter Van den Eede, Hong Nguyen Van, Chantal Van Overmeir, Indra Vythilingam, Thang Ngo Duc, Le Xuan Hung, Hung Nguyen Manh, Jozef Anné, Umberto D'Alessandro, Annette Erhart

**Affiliations:** 1 Department of Parasitology, Institute of Tropical Medicine, Antwerp, Belgium; 2 National Institute of Malariology, Department of Parasitology & Entomology, Hanoi, Vietnam; 3 Institute for Medical Research, Kuala Lumpur, Malaysia; 4 Katholieke Universiteit Leuven, Department of Microbiology and Immunology, Leuven, Belgium

## Abstract

**Background:**

Considering increasing reports on human infections by *Plasmodium knowlesi *in Southeast Asian countries, blood samples collected during two large cross-sectional malariometric surveys carried out in a forested area of central Vietnam in 2004 and 2005 were screened for this parasite.

**Methods:**

Blood samples collected at the 2004 survey and positive for *Plasmodium malariae *were randomly selected for PCR analysis detecting *P. knowlesi*. Blood samples collected in 2005 from the same individuals were screened again for *P. knowlesi*. Positive samples were confirmed by sequencing. Family members of positive cases who participated in both surveys were also screened.

**Results:**

Ninety-five samples with *P. malariae *mono- or mixed infections identified by species-specific PCR were screened for *P. knowlesi*. Among the five (5.2%) positive samples by PCR, three were confirmed to be *P. knowlesi *infections by sequencing, two young children (<5 years old) and a young man, all asymptomatic at the time of the survey and for the next six months after the survey. One of the two children was still positive one year later. No infection was found among the family members.

**Conclusion:**

*Plasmodium knowlesi *infections in humans can be found in central Vietnam. A small child was positive for *P. knowlesi *in both surveys at one year interval, though it is unclear whether it was the same or a new infection.

## Background

*Plasmodium knowlesi *has been recently defined as the "fifth human malaria species" [1] following the discovery in Malaysian Borneo of a large focus (58% of malaria cases in the Kapit hospital) of this simian malaria parasite in humans [2] and the more sporadic occurrence of other human cases in several Asian countries such as Thailand [3,4], Myanmar [5], The Philippines [6], and Singapore [7]. *Plasmodium knowlesi*, though usually found in long-tailed and pig-tailed macaques (*Macaca fascicularis *and *Macaca nemestrina*) in Southeast Asian (SEA) forested areas, can be naturally transmitted to humans by vectors belonging to the *Anopheles leucosphyrus *group, e.g. *Anopheles latens *in Malaysian Borneo and *Anopheles cracens *in Peninsular Malaysia [2,8,9]. *Plasmodium knowlesi*, genetically closely related to *Plasmodium vivax *[2,10], shares microscopically similarities with *Plasmodium malariae *and is characterized by a 24 h erythrocytic cycle. It may cause severe illness with risk of fatal outcome and it occurs without obvious clustering of cases in human settlements [2,11]. Known risk factors are adult age, forest-related activities or a recent travel history to forested areas [12-14]. Considering the increasing number of reports of human *P. knowlesi *infections in several SEA countries, blood samples collected during two large cross-sectional malariometric surveys carried out in 2004 and 2005 in a forested area in Central Vietnam were screened for the presence of this parasite.

## Methods

### Study area

The field study aiming at evaluating the effectiveness of long-lasting insecticidal hammocks for controlling forest malaria was carried out between 2004 and 2006 in Ninh Thuan province, located in the southern part of central Vietnam, in a population of about 20,000 people [15,16]. The study area is hilly and densely forested, inhabited mainly by the Ra-glai ethnic minority whose lifestyle is based on the exploitation of forest products, subsistence farming and cash crop cultivation. Beside their village homes, most families have plot huts in forest fields where they often stay overnight, particularly during the harvest season. Malaria transmission is perennial with two peaks (June and October) and is mainly supported by *Anopheles dirus sensu stricto *and *Anopheles minimus*, though several secondary vectors such as *Anopheles maculatus *and *Anopheles jeyporiensis *may be involved [17]. In 2004, the prevalence of malaria (all species) infection was 13.6% with a high proportion (>80%) of asymptomatic infections [16].

### Detection of malaria clinical cases and infections

Malaria incidence and prevalence were estimated by combining cross-sectional surveys and passive case detection (PCD) at village level. For the latter, since July 2004, febrile patients attending either the Commune Health Centers (CHC) or consulting the village health workers (VHW) were identified, had the body temperature and a blood sample taken for immediate diagnosis (rapid diagnostic test, RDT) and later microscopy. Patients were treated on the basis of the RDT results: *Plasmodium. falciparum *(including mixed infections) with a seven-day course of artesunate (16 mg/kg), and *P. vivax *with chloroquine (25 mg/kg) for three days [16]. In addition, following the trial design, a cohort of more than 4,000 randomly selected individuals was surveyed bi-annually, before and after the rainy season (April & December) [15,16]. At the time of the survey the body temperature was measured and the participants were interviewed about any symptoms in the previous 48 hours. Furthermore blood smears for microscopy and blood spots were collected on filter paper (Whatman N°3 filter paper) for later molecular analysis (species-specific PCR).

### Detection of *P. knowlesi *infections

Among the 210 *P. malariae *mono- or mixed infections identified by species-specific PCR on blood samples collected during the December 2004 survey, 95 were randomly selected to be screened for *P. knowlesi*. Forty-one of them were *P. malariae *mono-infections by species-specific PCR, while 54 were *P. malariae *mixed infections with either *P. falciparum *(15); *P. vivax *(15); *Plasmodium ovale *(5); *P. falciparum *and *P. vivax *(10); *P. vivax *and *P. ovale *(8); *P. falciparum*, *P. vivax *and *P. ovale *(1). By microscopy, 31 were negative, 42 mono-infections, i.e. 22 *P. falciparum*, 19 *P. vivax *and 1 *P. malariae*, and 11 mixed infections including two triple infections with *P. falciparum, P. vivax *and *P. malariae*. Family members of the *P. knowlesi *positive cases, included in the survey, were also screened for this parasite. Blood samples collected in December 2005 from the same individuals (index cases and their family members) were screened again for *P. knowlesi*.

Oral informed consent for blood sampling and malaria related analysis was obtained from all study participants after explanation of the study objectives and procedures in the local Ra-glai language.

### PCR

The DNA was extracted from filter paper with the Saponine-chelex method [18]. A nested PCR assay described by Singh *et al *[2] that specifically amplifies one part of the *P. knowlesi *small subunit ribosomal RNA (SSUrRNA) gene, was used to detect *P. knowlesi *DNA. Field samples confirmed (by species-specific PCR) to be mono-infections for *P. falciparum*, *P. ovale, P. malariae, P. vivax *[19], together with *P. knowlesi *positive samples from experimentally infected monkeys were used as controls. A negative control was included after 11 samples and at the end of each PCR. PCR conditions were as follows. The primary reaction was carried out in a 50 μl reaction mixture containing 1× reaction buffer (10× Qiagen Buffer), 3 mM MgCl_2 _(Qiagen, Hilden Germany), 200 mM of each deoxynucleoside triphosphate (Eurogentec, Belgium), 250 nM of each primers (rPLU1: 5'-TCA AAG ATT AAG CCA TGC AAG TGA-3'; rPLU5: 5'-CCT GTT GTT GCC TTA AAC TCC3'), 0.1 μg/μl acetylated BSA (Promega, Madison USA) and 1 U HotStarTaq Plus DNA polymerase (Qiagen, Hilden Germany) and 2 μl of DNA template was used for each reaction. The nested PCR amplification was carried out in a 25 μl reaction mixture containing 1× reaction buffer (10× Qiagen Buffer, Hilden Germany), 3 mM MgCl2 (Qiagen, Hilden Germany), 200 mM of each deoxynucleoside triphosphate (Eurogentec, Belgium), 250 nM of each primers (Pmk8: 5'-GTT AGC GAG AGC CAC AAA AAA GCG AAT-3'; Pmk9r: 5'-ACT CAA AGT AAC AAA ATC TTC CGT A-3') and 2 U HotStarTaq Plus DNA polymerase (Qiagen, Hilden Germany) and 2 μl of the primary PCR products were used as DNA templates. All PCR reactions were carried out using PTC 100 thermal cycler (Bio-Rad, California USA). 5 μl of the nested PCR products were loaded on a 2% agarose gel for 60 min at 5 V/cm using 0.5× TAE buffer. The gels were stained with ethidium bromide and visualized with UV.

### Cloning and sequence analysis

Before cloning, the PCR was repeated for the *P. knowlesi *positive samples to confirm the results. The nested PCR products of *P. knowlesi *samples were cloned using TOPO cloning kit, (Invitrogen, California USA). The PCR products were inserted in the pCR4^®^-TOPO plasmid and grown in *E. coli *(Mach1-T1R cells). Once cloned, they were purified using the quicklyse kit (Qiagen, Hilden Germany).

The cloned products were sequenced, and sequence alignment (with ClustalW [20]) was done with sequences of the 18S ribosomal sub-unit of *P. knowlesi *expressed during the sexual stages of the lifecycle or S-Type SSUrRNA gene, which were available in Genbank [GenBank accession numbers: DQ350263; DQ350262; DQ350261; DQ350260; DQ350259; DQ350258; DQ350257; DQ350256; DQ350255; DQ350271DQ350270 and U83876].

## Results

Ninety-five samples from the December 2004 survey were screened for *P. knowlesi*. Among the five (5.2%) initially found positive, only three remained positive after repeating the PCR. These were then confirmed by sequencing. One of the three index cases was re-confirmed to be positive one year later (December 2005 survey) for *P. knowlesi *(PCR and sequencing). The sample collected from this individual in 2004 had two sequences. The sequences (153 basepairs in size) obtained from the Vietnamese samples [GenBank accession numbers: FJ160750FJ160751; FJ160752; FJ160753 and FJ871986] showed 97-99% similarity with the Malaysian strains.

*Plasmodium knowlesi *infections were found in two young children (two-year old boy and three-year old girl) and a 27 years old man, all asymptomatic at the time of the survey. The microscopy examination had identified a *P. falciparum *and *P. vivax *mixed infection (3,120 parasites/μl) in the three-year old girl and a *P. vivax *mono-infection (32 parasites/μl) in the young man; the blood slide was negative in the other child. By species-specific PCR, all three individuals carried a mixed infection, the three-year old girl had *P. falciparum*, *P. vivax *and *P. malariae*, the two-year old boy had *P. malariae *and *P. ovale *and the young man *P. vivax *and *P. malariae*.

Six months prior to the survey, the passive case detection had identified a probable clinical attack (*P. falciparum *detected by RDT) in the adult man. No other malaria clinical attack among the *P. knowlesi *positive cases was identified by passive case detection during the entire study period (July 2004 to December 2006).

In the December 2004 survey, five out of the nine family members tested by PCR were positive for *P. knowlesi *infection, even after repeating such analysis. However, none of them was confirmed by sequencing. In the 2005 survey, the three-year old girl was again found positive by PCR for *P. knowlesi *and confirmed by sequencing, while the two other index cases and all concerned family members, were positive by PCR but were not confirmed by sequencing. The later results were due to aspecific reaction with human DNA, as the 161 basepairs fragment obtained by PCR displayed 97% similarity with a region of similar size of the human chromosome 16. The excess of human DNA and the possible binding sites for rPLU1 and rPLU5 were probably responsible for such aspecific reactions: rPLU1 and rPLU5 had respectively 80% and 76% similarity with their aspecific binding regions in the human genome.

All three confirmed *P. knowlesi *positive cases and their family members had a history of regular forest activities (children accompanying the adults), including nights frequently spent in the forest.

## Discussion

This is the first report of human *P. knowlesi *infections in Vietnam, not an unexpected finding considering the numerous reports from neighbouring countries [2-7]. Nevertheless, most previously reported cases were adults, often symptomatic, some of them with relatively high parasite densities. Instead, in this study, all *P. knowlesi *infections were asymptomatic, co-infected with *P. malariae*, with low parasite densities and two of the three identified cases were very young children under five years of age.

The *P. knowlesi *infections reported here were detected retrospectively by analysing blood spots on filter paper, thus the three individuals could not be followed up after the survey. Nevertheless, clinical malaria was carefully monitored in this population by passive case detection during which fever episodes were identified and checked for malaria infection. Though an undetected fever episode in the *P. knowlesi *infected individuals cannot be excluded with absolute certainty, none of the three *P. knowlesi *cases had any reported fever nor malaria-related consultation in the PCD during the whole study period (except the young adult man who was identified and treated for *P. falciparum *six months prior the 2004 survey). Moreover, during each of the subsequent surveys, none of the three patients had or reported fever.

One of the children still harboured the *P. knowlesi *infection one year later, though it is impossible to determine whether this was the same or a new infection. A possible approach would be the genotyping and comparison of both infections, though this would be helpful only if the parasite population was diverse enough. Finding an infection at one year interval in a young child indicates that in this area human exposure to *P. knowlesi *infection is not a rare event, either because the child may have been re-infected or because she was able to control the infection without developing symptoms, indicating some suppressing immunity.

The close contact of this community with the forest, where the *P. knowlesi *natural hosts can be found (monkeys are often kept as pets), is an additional element indicating a high risk of exposure to the infection. Indeed, the occurrence of *P. knowlesi *in SEA follows the distribution of its natural host, the long and pig tailed macaque [21], which mainly lives in the deep forest. Unlike other SEA countries, where recent economical development and deforestation have led to an increased invasion of the monkeys' habitat by man, in this Rag-lai community [22], the continuous contact with the forest probably means also a relatively constant exposure to *P. knowlesi *infection since the early years of life. Such exposure is made possible by the presence of an efficient vector that feeds both on humans and monkeys. The main malaria vector in central Vietnam, *An. dirus s.s*, belongs to the *An. leucosphyrus *group, which also includes the two confirmed vectors of *P. knowlesi *in Malaysia, *An. latens *and *An. cracens *[9,23,24]. *Anopheles dirus s.s *is a sylvatic species, usually highly anthropophilic which, in the absence of humans, can feed on monkeys especially in the canopy, and sporadically on other animals such as dogs, birds, and cattle [23]. Recently, *P. knowlesi *has been identified in an *An. dirus *specimen collected in central Vietnam (Khan Hoa province, neighboring the study area), indicating that *P. knowlesi *may be transmitted by this primary human malaria vector [25]. *Anopheles dirus s.s *ability to transmit *P. knowlesi *from monkey to monkey under lab conditions has already been reported [26], though the natural transmission from monkey to man has not been confirmed.

Confirmation of the *P. knowlesi *infection by sequencing was needed as the *P. knowlesi *PCR assay in this study had a low specificity, resulting in a substantial proportion of false positives. This is clearly illustrated by the high proportion of PCR positive results among the family members of the index cases later identified to be aspecific amplifications of human DNA. This also suggests that further improvement and standardization of the PCR technique is needed. Until there is no reliable and simple diagnostic test for detecting *P. knowlesi *infection, appreciating its distribution and burden in human populations will remain extremely difficult.

## Conclusion

*Plasmodium knowlesi *infection is present in Vietnam and has been found in a Rag-lai community living in a relatively remote area in close contact with the forest. Two of the three identified infections occurred in young children below five, and in one of them, *P. knowlesi *was identified again one year later, indicating that human exposure to *P. knowlesi *infection is not a rare event. Interestingly, none of the *P. knowlesi *positive cases had malaria clinical symptoms either during the surveys, or during passive case detection until one year after each survey.

A better estimation of the importance of *P. knowlesi *infection in this population, as well as in other forested areas in central Vietnam would need larger studies. However, considering the difficulties in diagnosing *P. knowlesi *infections, simpler and more specific diagnostic tools are urgently needed.

## Conflict of interests statement

The authors declare that they have no competing interests.

## Authors' contributions

PVDE carried out the PCR analysis, data analysis and paper writing. HNV and CVO helped in the PCR analysis. IV kindly provided the training and expertise for the set up of the *P. knowlesi *at the Department of Parasitology, ITM Antwerp. AE and UDA made substantial contributions to conceive the study design, paper writing and reviewing. NDT, LXH, and AE coordinated the cluster randomized trial in Ninh Thuan province. NMH and JA revised the manuscript. All authors red and approved the final manuscript
